# Évolution de leucémies myéloïdes chroniques sous nilotinib après échec a l'imatinib

**DOI:** 10.11604/pamj.2014.18.142.3308

**Published:** 2014-06-17

**Authors:** Salifo Sawadogo, Francis Michel Hien, Macaire Sampawendé Ouédraogo, Youssouf Joseph Drabo

**Affiliations:** 1 Laboratoire d'hématologie et d'immunologie du Centre hospitalier universitaire Sourô Sanou, 01 BP 676 Bobo-Dioulasso 01 Burkina Faso; 2 Service de médecine interne, Centre hospitalier universitaire Sourô Sanou, 01 BP 676 Bobo-Dioulasso Burkina Faso; 3 Service de médecine interne Centre hospitalier universitaire Yalgado Ouédraogo, 03 BP 7022 Ouagadougou 03 Burkina Faso

**Keywords:** Leucémies myéloïdes chroniques, Imatinib, résistance, intolérance, Nilotinib, chronic myeloid leukemia, Imatinib, drug resistance, intolerance, Nilotinib

## Abstract

C'est une étude observationnelle prospective ouverte: quatre leucémies myéloïdes chroniques résistant ou intolérant à l'Imatinib ont été traitées par le Nilotinib. Elles ont été incluses dans le programme GIPAP et suivies selon les recommandations de “European LeukemiaNet”. Trois ont un score de Sokal de haut risque et une de bas risque. Deux étaient hypertendues. Mises sous Nilotinib, il y a eu deux rémissions cytogénétiques complètes et deux échecs. Le traitement a été interrompu chez les deux rémissions complètes, l'un pour effet secondaire du Nilotinib et l'autre pour changement de pays. Les deux échecs sont dus à des résistances. Le Nilotinib réduisant la fréquence des mutations des leucémies myéloïdes chroniques à haut risque et risque intermédiaire, il serait judicieux d'utiliser ce produit en première intention dans ces cas - ci pour réduire la charge des examens complémentaires. Les pays à bas revenu confrontés à des problèmes de survie ont besoin de la solidarité mondiale pour prendre en charge les leucémies myéloïdes chroniques.

## Introduction

Le traitement de la leucémie myéloïde chronique a été radicalement amélioré avec l'introduction des anti- tyrosines kinases dont le chef de file est l'Imatinib. Des échecs sont rencontrés toutefois avec cette nouvelle molécule. Les mécanismes de ces échecs sont dus: -soit à des résistances primaires ou secondaires dépendant des mutations de BCR-ABL; -Soit à des mécanismes indépendants de BCR-ABL; -soit à une intolérance au médicament [1]. Le Nilotinib est une anti-tyrosine kinase de deuxième génération indiquée dans le traitement des leucémies myéloïdes chroniques en phase chronique ou accélérée après échec à l'Imatinib ou au Dasatinib [2]. Le Nilotinib est aussi utilisé en première ligne de traitement dans cette maladie parce qu'il réduit la fréquence des mutations de BCR-ABL [3]. Nous rapportons quatre cas traités au Nilotinib après échec à l'Imatinib au Centre hospitalier universitaire Sourô Sanou.

## Patient et observation

Quatre patients dont trois hommes et une femme ont été inclus dans le programme international Glivec d'aide aux patients.

C'est une étude observationnelle ouverte et prospective. Chaque patient a reçu le Nilotinib (Switzerland: Novartis Pharma) à la posologie de 800mg/j en deux prises après un repas. La surveillance a été faite selon les recommandations de “European LeukemiaNet” [4, 5].

**Le premier patient** âgé de soixante trois ans au diagnostic était hypertendu, avec un score de Sokal de bas risque et une translocation génétique classique. Il a été traité par l'hydroxyuré pendant 51 mois puis par l'Imatinib (Switzerland: Novartis Pharma) à raison de 400mg/J. Il a été mis en rémission cytogénétique complète après onze mois de traitement. Cette rémission cytogénétique complète a été perdue après 51 mois de suivi, sans progression clinique et hématologique. L'augmentation de la posologie à 600mg /j pendant quatorze mois n'a rien changé sur le plan cytogénétique. Après la mise sous Nilotinib pendant 25 mois après l'Imatinib, il n'y a pas eu de progression hématologique; mais aucune réduction du chromosome Philadelphie n'a été notée. En raison d'un trouble de la repolarisation et d'une augmentation de QTc au-delà de 450 ms sur l’électrocardiogramme, le traitement a été interrompu [6].

**Le deuxième patient** avait vingt quatre ans au diagnostic. Découvert à la suite d'un priapisme, il avait un score de Sokal de haut risque et une translocation complexe impliquant les chromosomes 9.17 et 22. Il a été traité pendant 22 mois avec une rémission cytogénétique partielle au bout de 13 mois qui a été perdue trois mois après. L'augmentation de la posologie n'a pas empêché la progression avec apparition d'une trisomie 8 et une duplication du chromosome Philadelphie. A la suite du traitement au Nilotinib, une rémission cytogénétique complète a été notée quatre mois après le début et une rémission moléculaire partielle 7 mois après. Le traitement a duré 30 mois et la prise du médicament a été interrompue parce que le patient a changé de pays.

**Le troisième patient** était âgé de dix sept ans au diagnostic, avec un score de Sokal de haut risque et une translocation classique. Il y a eu une rémission cytogénétique partielle au bout de huit mois de traitement avec l'Imatinib. Cette rémission a été totalement perdue six mois après et l'augmentation des doses pendant 32 mois n'a rien changé sur le résultat cytogénétique. Il a été traité pendant 14 mois avec le Nilotinib après l'arrêt de l'Imatinib mais le chromosome Philadelphie était toujours à 100%. Traité par l'Omacétaxine Mépésuccinate par la suite, [7] le traitement a été interrompu en raison de la survenue d'une thrombopénie de grade 4 que notre système transfusionnel actuel ne peut manager sans risque.

**Le quatrième patient** était une femme de quarante quatre ans, sous traitement avec l'hydroxyuré ainsi qu'avec l'Imatinib. Elle a présenté une leuco-neutropénie de grades 3 et 4 avec des nouures musculaires et une thrombopénie de grade 4 avec cécité bilatérale par hémorragie rétinienne bilatérale. Son score de Sokal était de haut risque et elle était hypertendue. Elle a été traitée avec le Nilotinib pendant 45 mois et la rémission cytogénétique complète a été constatée 30 mois après le début. Le traitement a été arrêté en raison du développement d'une cardiopathie congestive.

## Discussion

Le Nilotinib a été utilisé en deuxième ligne de traitement et, selon les recommendations de Leukemia Net les indications ont été: trois résistances à l'Imatinib sans perte de la réponse hématologique et une intolérance à l'Imatinib sans aucune réduction du PH+. Les mutations de BCR-ABL ont été constatées plus chez les patients ayant un score de Sokal de haut risque ou intermédiaire [3]. Trois des patients ont présenté un score de haut risque, mais pour des raisons financières, aucun séquençage de BCR-ABL n'a été réalisé pour rechercher une éventuelle mutation BCR-ABL avant la prescription du Nilotinib. Les étiologies d'une résistance indépendante de BCR-ABL n'ont pas non plus été explorées. Les résultats cliniques hématologiques et cytogénétiques ont servi de guide de définition des indications. Dans la littérature médicale [5)], 56% des patients résistant à l'Imatinib et 66% des patients intolérant à l'Imatinib ont une réponse cytogénétique majeure; 41% des résistants à l'Imatinib et 51% des intolérants à l'Imatinib ont une réponse cytogénétique complète.

Sur les quatre patients, deux sont en rémission cytogénétique complète: un résistant à l'Imatinib et un intolérant à l'Imatinib. Les deux autres, toujours en phase chronique, sont en échec cytogénétique; aucune réduction du Ph+ malgré l'augmentation des doses et la durée du traitement.

Deux des patients étaient hypertendus: l'un résistant à l'Imatinib, sans rémission cytogénétique sous Nilotinib et l'autre intolérant à l'Imatinib avec rémission cytogénétique sous Nilotinib. Les effets secondaires du Nilotinib excluant l'utilisation de ce produit ont été la cause de l'interruption du traitement chez ces patients. Aucune alternative thérapeutique n'a pu être proposée pour des raisons financières.

Pour les deux autres patients, l'interruption a été due à une perte de vue chez l'un, après une rémission cytogénétique complète et une rémission moléculaire majeure Chez l'autre; l’échec sous Nilotinib a été la cause de l'interruption. Dans les pays à faible ou moyen revenu, le problème de la prise en charge des leucémies myéloïdes chroniques n'est pas lié à une connaissance de meilleures stratégies thérapeutiques mais plutôt à un problème de disponibilité de molécules et d'examen de compléments adaptés. La tendance actuelle est d'utiliser le Nilotinib en première intention de traitement dans la LMC du fait de son efficacité à réduire plus la survenue des mutations par rapport à l'Imatinib [8]. Stratégiquement, il serait mieux pour les pays à faible revenu et qui ont des difficultés à rechercher les mutations dues au traitement d'utiliser le Nilotinib en première intention pour le traitement des Leucémies myéloïdes chroniques.

## Conclusion

Le traitement des leucémies en général et de la leucémie myéloïde chronique en particulier restera comme une symphonie inachevée tant que la cellule souche leucémique restera inaccessible au traitement. Les pays à faible revenu confrontés à des problèmes de survie ont besoin de la solidarité mondiale pour la prise en charge de ces malades.
